# Participatory development of inclusive health communication on COVID-19 with homeless people

**DOI:** 10.1093/eurpub/ckac130.237

**Published:** 2022-10-25

**Authors:** A Specht, N Sarma, T Hellmund, T Linzbach, M Hörig, M Wintel, FP Mockenhaupt, J Seybold, AK Lindner

**Affiliations:** 1 Institute of Tropical Medicine and International Health, Charité - Universitätsmedizin Berlin, Berlin, Germany; 2 Department of Infectious Disease Epidemiology, Robert Koch Institute, Berlin, Germany; 3 Medical Directorate, Charité - Universitätsmedizin Berlin, Berlin, Germany

## Abstract

**Background:**

The COVID-19 pandemic demonstrates the important role of providing people with easy to access up-to-date health information in digital formats. People experiencing homelessness have limited access to health information and were hardly ever directly addressed through communication channels in Germany. Lack of digitalization within shelters and social services, as well as technical and socio-economic barriers in purchasing and maintaining a smartphone are further barriers to information.

**Methods:**

The Charité-COVID-19-project for and with homeless people has created digital health information videos and posters, with an interdisciplinary team and in a participatory approach. Two videos on general information and testing of COVID-19 were launched in 5 languages in February 2021. Vaccination posters in two language versions including 9 languages are available since April 2021.

**Results:**

We will present the collaboration of research, practice and community, the production process, the distribution and the acceptance of the formats. The web link refers to the videos, posters and further information:

https://tropeninstitut.charite.de/forschung/charite_covid_19_projekt_fuer_und_mit_obdachlosen_menschen/

**Conclusions:**

Exclusion from (digital) information is an increasingly important part of the structural marginalization of homeless people. This, as well as the inadequate consideration of this population in health communication and the pandemic response have to be addressed. Tackling the digital gap allows improved access to health information for homeless people and promotes health-seeking behaviour. Empowerment of the community through participation and a network between community, service providers, politics and research are also crucial for improvement of homeless people's health in the future.

**Key messages:**

- Exclusion from (digital) information is an increasingly important part of the structural marginalization of homeless people that has to be addressed.
- Bridging the gap between research, practice and community was a key factor for the realisation of inclusive digital health communication with homeless people in this project.

